# Characterizing Mechanical Changes in the Biceps Brachii Muscle in Mild Facioscapulohumeral Muscular Dystrophy Using Shear Wave Elastography

**DOI:** 10.3390/diagnostics14171985

**Published:** 2024-09-08

**Authors:** Benedict Kleiser, Manuela Zimmer, Filiz Ateş, Justus Marquetand

**Affiliations:** 1 Department of Epileptology, Hertie-Institute for Clinical Brain Research, University of Tübingen, Hoppe-Seyler-Str. 3, 72076 Tübingen, Germany; 2 Department of Neural Dynamics and Magnetoencephalography, Hertie-Institute for Clinical Brain Research, University of Tübingen, Otfried-Müller-Str. 25, 72076 Tübingen, Germany; 3 MEG-Center, University of Tübingen, Otfried-Müller-Str. 47, 72076 Tübingen, Germany; 4 Institute of Structural Mechanics and Dynamics in Aerospace Engineering, University of Stuttgart, Pfaffenwaldring 27, 70569 Stuttgart, Germany; 5 Institute for Modelling and Simulation of Biomechanical Systems, University of Stuttgart, Pfaffenwaldring 5a, 70569 Stuttgart, Germany

**Keywords:** SWE, FSHD, ultrasound elastography, sEMG, skeletal muscle mechanics

## Abstract

There is no general consensus on evaluating disease progression in facioscapulohumeral muscular dystrophy (FSHD). Recently, shear wave elastography (SWE) has been proposed as a noninvasive diagnostic tool to assess muscle stiffness in vivo. Therefore, this study aimed to characterize biceps brachii (BB) muscle mechanics in mild-FSHD patients using SWE. Eight patients with mild FSHD, the BB were assessed using SWE, surface electromyography (sEMG), elbow moment measurements during rest, maximum voluntary contraction (MVC), and isometric ramp contractions at 25%, 50%, and 75% MVC across five elbow positions (60°, 90°, 120°, 150°, and 180° flexion). The mean absolute percentage deviation (MAPD) was analyzed as a measure of force control during ramp contractions. The shear elastic modulus of the BB in FSHD patients increased from flexed to extended elbow positions (e.g., *p* < 0.001 at 25% MVC) and with increasing contraction intensity (e.g., *p* < 0.001 at 60°). MAPD was highly variable, indicating significant deviation from target values during ramp contractions. SWE in mild FSHD is influenced by contraction level and joint angle, similar to findings of previous studies in healthy subjects. Moreover, altered force control could relate to the subjective muscle weakness reported by patients with dystrophies.

## 1. Introduction

Facioscapulohumeral muscular dystrophy (FSHD) is a genetic muscular dystrophy that typically affects shoulder and proximal arm muscles, leading to muscle paresis. Until now, there has been no general agreement on measuring disease progression or treatment effects of the different new therapeutic approaches and taking biomechanical changes in the muscle into account. Conventionally, along with questionnaires and scales, such as the Facioscapulohumeral Muscular Dystrophy-Health Index [1] or the measurement of muscle force by torque [2], the extent of ongoing dystrophy in the muscle is estimated by various imaging options, such as conventional ultrasound or magnetic resonance imaging (for a review, see [3]). B-mode ultrasound can indicate changes in muscle architecture, resulting in muscle echogenitiy [4,5]. The structural changes, which histopathologically correspond to atrophy, fibrous transformation, and the fatty infiltration of the musculature identified in muscular dystrophies [6,7], suggest alterations in mechanical properties, such as muscle stiffness. The resulting functional impairment in patients with FSHD can be quantified with measures of muscle strength or movement tests, such as evaluating the 3D reachable workspace [8]. Ultrasound shear wave elastography (SWE) poses a promising imaging modality for assessing muscle stiffness (e.g., [9,10]). It provides a shear elastic modulus value as a measure of tissue stiffness by assuming a transverse isotropic, linear elastic material characteristic for muscle tissue [9,11]. Previous studies have related changes in the shear elastic modulus with muscle force (e.g., measured by the resulting joint moment [9,11,12,13]), demonstrating the potential of SWE to characterize muscle function. Thus, in patients with FSHD, SWE could offer a measure of altered muscle mechanics due to a dystrophic transformation in passive and active states (i.e., during different contraction levels), as muscle changes in the passive state were already observed by SWE in other muscle diseases (e.g., [10,14]).

SWE has been used for other dystrophic diseases, such as Duchenne dystrophy [10,15,16,17] and Fukuyama-type congenital muscular dystrophy [18]. In both dystrophies, the shear elastic modulus of affected biceps and triceps brachii muscles was reported to be higher than those of a healthy group during rest but has not been studied during muscle contraction. Characterizing muscle mechanics in relation to joint moment as an estimate of muscle force production capacity could be a promising biomarker for dystrophies. Although SWE has been used to describe structural and functional changes in diseased muscles (e.g., [10,14]), the relationship between disease progression and muscle mechanical adaptation has not yet been established. Since force production of a muscle is dependent on its length and activation level [19], joint position and contraction intensity affect its tensile load [19] and stiffness [20]. Previous studies have elaborated that these influencing factors can be detected from the measured shear wave velocity (SWV) [11]. For example, even low-level muscle activation that can be observed during rest [20,21] may result in significant changes in the measured output, yet many studies have not considered this [22]. Moreover, different muscle groups showed different values of muscle stiffness [23]. These factors can lead to low reproducibility and reliability of SWE if they are not carefully controlled in the study setup, which may explain why muscle SWE has not yet been used in clinical routine diagnostics of muscle diseases. Therefore, we decided to use a study setting with exact control regarding the joint position, especially in the elbow, the measurement of a muscle with homogeneous parallel muscle fibers, and a parallel recording of the muscle activity with surface electromyography (sEMG).

Here, we aimed to comprehensively study the diseased muscle in patients with mild FSHD at different muscle lengths and contraction intensities. The purpose of this study was to characterize the stiffness of diseased biceps brachii (BB) muscle, both in a passive state (i.e., during rest) and in an active state (i.e., during isometric contraction), while simultaneously measuring elbow moment and muscle activity using sEMG. We hypothesized that (1) the shear elastic modulus increases with increasing joint angle and contraction intensity and (2) the functional changes in the muscles during contraction also lead to different changes in SWE in the active state.

## 2. Materials and Methods

### 2.1. Participants

In this cross-sectional pilot study, eight patients with genetically confirmed FSHD were recruited from the neurological department of the University Hospital of Tübingen and through the Diagnosegruppe FSHD of the Deutsche Gesellschaft für Muskelerkrankte e.V., a German association of individuals with muscle diseases. The measurements were compared with those of a healthy group (HG) published elsewhere [11]. Any potential differences from the FSHD group will be addressed as needed in the following Materials and Methods section. As an inclusion criterion, patients with FSHD were required to have a self-reported loss in strength in elbow flexion. Exclusion criteria were applied if patients could not maintain their arms in front of their bodies or perform elbow flexion against gravity (a minimum Medical Research Council (MRC) score [24,25] of ≥4−/5 for elbow flexion). This exclusion ensured that patients could effectively engage in the experimental paradigm. The measurement setup was designed to be wheelchair accessible, and all measurements were conducted on the dominant arm according to the participants’ handedness. The upper arm circumference was measured at rest with the elbow extended. The participants were instructed to avoid extensive exercise or muscle loading in the days preceding the measurement. This study received approval from the local ethics committee and adhered to the principles outlined in the Declaration of Helsinki (Ethics Committee at the Medical Faculty of the Eberhard Karls University and at the University Hospital of Tübingen, project number 612/2021BO2, 15 September 2021 and 22 October 2021). Informed consent was obtained from all the participants.

### 2.2. Measurement Setup

**Figure 1:**
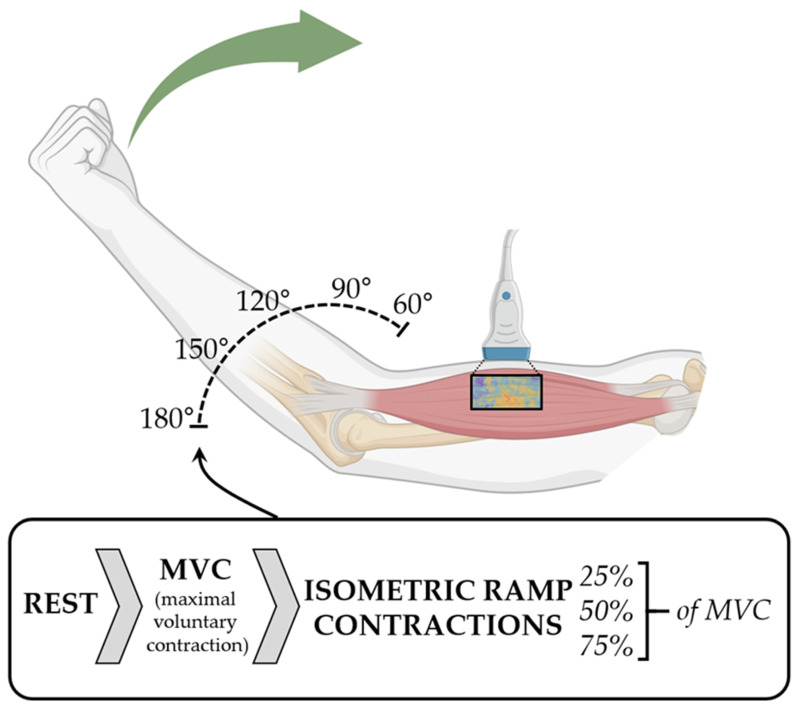
Illustration of the measurement setup. Shear wave elastography of the biceps brachii muscle was performed at rest, and maximal voluntary contraction (MVC) and ramp contractions (up to 25%, 50%, and 75% MVC elbow moment) were performed at different elbow angles (60°, 90°, 120°, 150°, and 180°). Created with BioRender.com.

A custom-built elbow moment measurement system was used [11]. The participants were asked to sit upright with one elbow positioned on a height-adjustable pillar in front of them so that the shoulder was flexed at 90°. Five different elbow angles (60°, 90°, 120°, 150°, and 180°) were investigated (Figure 1), and the subjects were asked to close a hand at a handhold on the lever while the wrist was in a supinated position and was stabilized with a wrist protector.

### 2.3. Measurement Protocol

The data collection began with a warm-up protocol, including one maximal voluntary isometric elbow flexion and four submaximal ramp contractions (in HG: six submaximal ramp contractions) to familiarize the participants with the experimental tasks. After that, maximal voluntary isometric elbow extension was performed at a 120° elbow angle (in FSHD, once, and in HG, three times). Then, for each elbow joint position (60°, 90°, 120°, 150°, and 180°), 5 s during rest (i.e., a relaxed muscle state) was recorded. Afterward, the participants were asked to contract maximally (isometric elbow flexion) for 5 s (in FSHD, once, and in HG, three times). The MVC elbow moment was calculated and used for the normalization of isometric ramp contractions up to 25%, 50%, and 75% MVC elbow moment (five parts: 3 s rest, increase with 6.25% per second slope, plateau phase with the required percentage of MVC for 5 s, decrease 6.25% per second slope, 3 s rest). The participants were asked to follow a line visualizing their elbow moment in real-time to perform these ramp contractions. The 25%, 50%, and 75% MVC elbow moment plateau phase lasted for 5 s. Three ramp trials were conducted for healthy subjects at each level and position. After completing the protocol validation in healthy subjects, the number of trials for FSHD patients was reduced to just one trial per level and position to minimize the overall length for patients. After MVC trials, FSHD patients had a minimum pause of 2 min, after ramp contractions of 1 min to reduce the potential risk of fatigue during the protocol (for HG: after MVC trials, 1 min rest; after each ramp, 30 s).

### 2.4. Equipment

#### 2.4.1. Elbow Flexion Moment

A static torque sensor (DF30, 200Nm, Lorenz Messtechnik GmbH, Aldorf, Germany) was used to measure the elbow moment. The mean absolute percentage deviation (MAPD) between the applied and required elbow moments was calculated for the ramp contractions’ middle three seconds of the plateau phase. Trials with MAPD > 10% were excluded from further analysis (including SWE and further MAPD calculations) to exclude trials that did not reach the demanded force level due to errors of the participants (e.g., impaired concentration in one specific trial). To evaluate the force control, the MAPD was calculated for the performance of the ramp (whole plateau phase, including the increase and decrease in elbow moment to reach and to leave the plateau phase).

#### 2.4.2. Sonography and Shear Wave Elastography (SWE) of the Biceps Brachii

SWE and sonography were performed using the AixPlorer Mach30 (MSK preset; Supersonic Imagine, Aix-en-Provence, France). For the SWE measurements, the ultrasound probe (5–18 MHz, linear-array, 50 mm wide; L18–5; Supersonic Imagine, Aix-en-Provence, France) was placed in the middle of the upper arm on the BB in a longitudinal direction (aligned with muscle fiber direction; see Figure 1). The region of interest was placed at a maximal depth of 4 cm, and the maps of the shear elastic modulus was recorded at 1–1.8 Hz, using a maximal range of 200 kPa (14.1 m/s for SWV). The shear elastic modulus was calculated from the measured SWV assuming transversely isotropic, lossless linear elastic material characteristics in an unloaded condition. The mean shear elastic modulus in the ultrasound frames was analyzed if no more than 25% of the pixels inside the chosen region of interest were missing. All available frames were used for the calculation of shear elastic modulus at rest and MVC. The frames of the ramps were resampled to 1 Hz from their original SWE sampling rate, and the mean was calculated from the middle three seconds of the plateau phase when the subjects best followed the prescribed line.

Furthermore, dimensions of the biceps brachii muscle (BB) were taken during rest for all elbow positions. The insertion and origin of the short head of the BB were sonographically detected in the longitudinal plane. The length of the muscle was then measured on the skin using a tape measure. From B-mode ultrasound images in a transverse plane at the approximate location of the maximum muscle belly area (ultrasound probe upright on the forearm at the maximum belly at the middle of the forearm), the cross-sectional area (CSA) was measured by marking the muscle fascia of the BB.

#### 2.4.3. Surface Electromyography of the Biceps Brachii (BB) and the Triceps Brachii

Surface electrodes of the Trigno Wireless Biofeedback System (Delsys Europe, Greater Manchester, UK) were used to monitor the electromyographic activity of the BB and the triceps brachii (TB). A fourth-order Butterworth filter (20–350 Hz bandwidth) and full-wave rectification were applied to the sEMG data as typically recommended [26], and the root-mean-square moving average (sEMG-RMS) with a window size of 250 ms was calculated.

The SENIAM recommendations for electrode placement were considered [27]. Two electrodes (Trigno Mini Sensors) were placed slightly laterally and medially to the central BB position so that the ultrasound probe could be placed in between. Two other electrodes (Trigno Duo Sensor) were placed on the lateral and long head of the TB. The averages of the two BB and two TB electrodes represented the BB and TB, respectively.

The sEMG-RMS during rest were normalized to the MVC measurements (for the BB: MVC flexion at each angle, for the TB: MVC extension at 120°). A 75% ramp was used to normalize both muscles at each angle for the isometric ramp contractions. Trials during rest with the sEMG-RMS >5% were not considered to represent the passive muscle state and were excluded from further analyses.

#### 2.4.4. Data Acquisition

Elbow moment and sEMG data were recorded simultaneously using a data acquisition system (cDAQ-9174, National Instruments, Austin, TX, USA, sampling rate of 2 kHz). Ultrasound videos were triggered and recorded simultaneously using an Arduino board (Arduino S. r.l., Monza, Italy).

### 2.5. Statistics

Statistics were calculated using the Statistical Package for the Social Sciences (SPSS, version 27 and 29 for Windows, IBM Cooperation, Armonk, NY, USA). JMP (Version 16.2, SAS Institute Inc., Cary, NC, USA, 1989–2023) was chosen for creating the boxplots. Due to the low sample size (*n* = 8), normal distribution could not be assumed. The Friedmann test investigated within the FSHD group the effects of the elbow angle on elbow moment and on SWE as well as the effects of the force level on SWE. Furthermore, the effects of the angle on the muscle length and CSA were analyzed using the Friedman test. Significant differences within the FSHD group were confirmed post hoc by Dunn–Bonferroni tests. To compare SWE and MAPD values presently collected from the FSHD group with the previously published HG [11], we used the Mann–Whitney U test for each single condition for angle and force level (15 conditions for MAPD and 25 conditions for SWE), and to avoid p-hacking, multiple testing was performed using the correction of Benjamini and Hochberg, also named the False Discovery Rate.

## 3. Results

### 3.1. Participants, Anthropometrics, and Elbow Moment Performance

Eight patients with genetically confirmed FSHD were included and compared to fourteen healthy volunteers (see Table 1 for demographic details). The patients with FSHD showed different levels of weakness according to the MRC [24,25] but not less than 4− due to the exclusion criteria and different alterations in the B-mode ultrasound image of the BB classified by the modified Heckmatt scale [28] (see Table 2).

The length and CSA of the BB are given for every angle in Table 3. The elbow moment decreased with increasing angle (*p* < 0.001). Post hoc tests indicated significant differences between 60° (*p* = 0.016), 90° (*p* = 0.027), 120° (*p* < 0.001), and 180°, as well as 120° and 150° (*p* = 0.016). Four passive-state trials in the FSHD group were excluded from further analysis of the passive muscle state due to the sEMG-RMS being > 5% during rest (one trial at 90°, one trial at 120°, and two trials at 150°). Four ramp trials with MAPD > 10% in the middle three seconds of the plateau phase were excluded from further analysis (one trial for 75% MVC at 120°, two trials for 75% MVC at 150°, and one trial for 75% MVC at 180°).

### 3.2. MAPD

**Figure 2:**
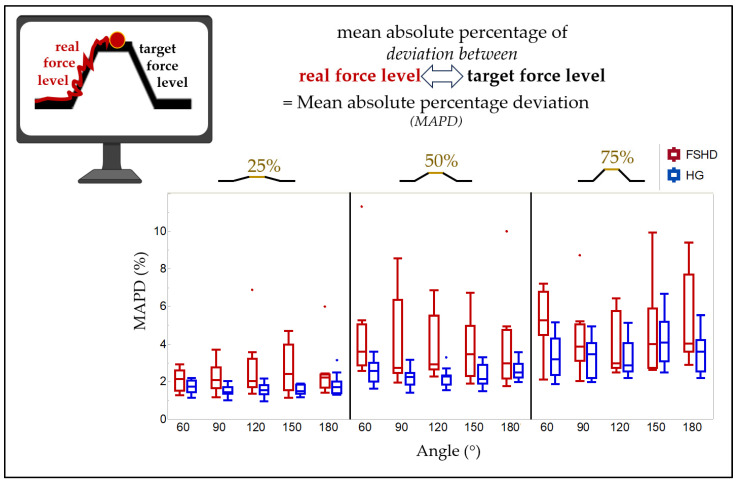
Boxplots of the mean absolute percentage deviation (MAPD) during isometric ramp contractions at different elbow angles (60°, 90°, 120°, 150°, and 180°) and ramp levels (25%, 50%, and 75% of MVC), shown for the FSHD group and the healthy group (HG).

The respective MAPD of the ramp contraction (including force increase, plateau phase, and force decrease) was highly variable (see Figure 2, Table A1). It increased with rising contraction intensity from 25% MVC to 75% MVC (e.g., at 60° (*p* < 0.001), post hoc: 25% MVC and 50% MVC (*p* = 0.018), 25% MVC and 75% (*p* = 0.003)). There was not an obvious influence of the angle in FSHD patients (at 25% MVC, *p* = 0.981; at 50% MVC, *p* = 0.357; at 75% MVC, *p* = 0.027; post hoc: no significant differences between the individual positions).

### 3.3. SWE

**Figure 3:**
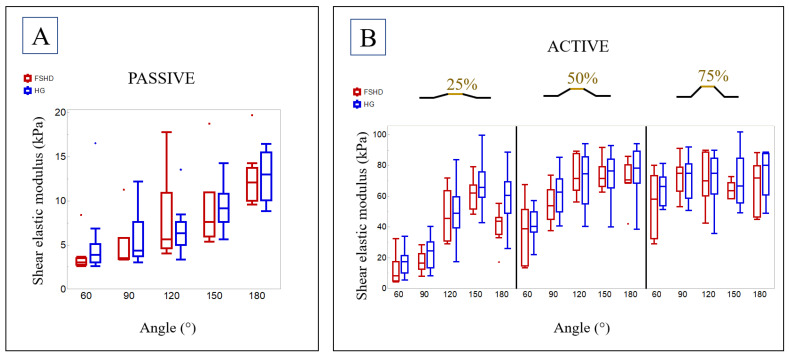
Boxplots for the shear elastic modulus at different elbow angles (60°, 90°, 120°, 150°, and 180°) shown for the FSHD group and the healthy group (HG) (**A**) at passive state and (**B**) at different ramp levels (25%, 50%, and 75% of MVC).

The passive-state shear elastic modulus increased with increasing elbow angle from flexion to extension (60° to 180°, *p* = 0.003, Figure 3A). Post hoc analysis indicated significant differences between 60° and 180° (*p* = 0.019). For example, the median shear elastic modulus at 90° was 3.5 kPa (range 3.3 kPa–11.3 kPa) and 12.1 kPa (9.6 kPa–19.7 kPa) at 180°. Likewise, shear elastic modulus increased with increasing elbow angle from flexion to extension at 25% MVC (*p* < 0.001, post hoc: 60° and 120° (*p* = 0.044), 150° (*p* < 0.001), 180° (*p* = 0.009) as well as 90° and 150° (*p* = 0.001)) and at 50% MVC (*p* < 0.001, post hoc: 60° and 120° (*p* = 0.016), 150° (*p* = 0.009), 180° (*p* = 0.016); see Figure 3B). During MVC (*p* = 0.882) and 75% MVC (*p* = 0.090), no significant differences could be found between the different angles.

The shear elastic modulus extracted from the plateau phases of the ramp contractions increased significantly with rising contraction intensity from passive state to MVC in every investigated angle (e.g., at 60° (*p* < 0.001), post hoc: passive state and 75% MVC (*p* < 0.001), MVC (*p* < 0.001); 25% MVC and 75% MVC (*p* = 0.044), MVC (*p* = 0.005)).

### 3.4. Comparison with a Healthy Group

In regards to the comparison with the previously published HG (n = 14, Table 1) [11], for the trials in HG, two passive-state trials (120° and 150°), five ramp trials (two for 75% MVC at 60°, two for 75% at 120°, one for 75% at 180°), and two SWE measurements (one ramp for 25% MVC at 90°, one MVC at 60°) were excluded. There was no significant difference in the elbow moment at MVC between the FSHD group and the HG (60°: *p* = 0.110, 90°: *p* = 0.297, 120°: *p* > 0.999, 150°: *p* = 0.714, 180°: *p* = 0.868). However, patients with FSHD deviated from the target value during ramp contractions significantly more than the HG and after correction for multiple testing especially at 50% of MVC (see Figure 2, Table A1). The median MAPD during ramp contractions for 50% MVC at 120° was 2.9 percentage points for the FSHD group (range 2.3–6.9) and 2.3 for the HG (range 1.5–3.3).

For SWE, there was a significant difference between FSHD and HG only during 25% MVC at 180° (*p* = 0.006); however, it was not significant after correcting for multiple testing (*p* = 0.150) (see Table A2). All the other conditions in passive and active state show no significant differences.

## 4. Discussion

This study comprehensively investigated the mechanical properties of the BB using SWE in mildly affected patients with FSHD and yielded three main findings:

(1) In muscle dystrophies such as FSHD, the shear elastic modulus depends on the joint angle and the percentage of muscle activation.
(2) In contrast to previous studies on dystrophic muscle diseases [10,15,16,18,29], the shear elastic modulus in a passive state was not higher in the mild-FSHD group than in the HG, but careful considerations have to be made before concluding a potential non-existing difference between healthy and dystrophic altered muscles (see below).
(3) Patients with FSHD could exhibit a lack of force control, indicated by a higher MAPD; in other words, they possibly deviated significantly more from the elbow moment they were instructed to follow during the ramp task than the HG did.

In the following sections, we elaborate on the use of SWE and MAPD, potentially representing disease-related neuromuscular alterations in the FSHD group.

### 4.1. Shear Elastic Modulus in FSHD

In this pilot study, we aimed to characterize altered active muscle function in muscle dystrophies, such as FSHD, using SWE. For the FSHD group, we observed an increase in the shear elastic modulus of the BB with rising ramp level and extension in the elbow. Previous studies on SWE during muscle contraction have demonstrated an increase in the shear elastic modulus with increasing contraction intensity in healthy subjects [11,13,30,31,32], suggesting that the shear elastic modulus could be used as a surrogate for muscle force. However, those studies are limited to the shear elastic modulus–force relationships for an individual muscle (e.g., [13]) or a homogeneous group (e.g., [11]). Recent studies have attributed changes in the measured shear elastic modulus in an active muscle state to both changes in muscle force production and muscle stiffness [33,34]. In mild FSHD, we also observe that the shear elastic modulus depends on the force level, suggesting that SWE could also represent muscle force as in healthy subjects. Interestingly, the shear elastic modulus in some 75% MVC trials was even higher than in the MVC trials, which was already observed in healthy subjects [11]. This may be attributed to the different contraction types (slow submaximal contraction vs. short maximal contraction) [11]. Therefore, further investigation of SWE in the different contraction types in patients and matched healthy controls is needed to understand the possible influence better.

Furthermore, in FSHD, the shear elastic modulus increases with rising extension in the elbow, particularly in passive state and at 25% and 50% of MVC, while no significant differences could be observed at MVC and 75% of MVC. In general, the influence of the joint position on the shear elastic modulus is common, especially at rest and in passive state (e.g., [12,23]), and is also described in other muscle dystrophies as Duchenne [29] or muscle diseases like inclusion body myositis [14] at rest. Interestingly, as already observed in healthy subjects, at low-level contractions (e.g., 25% MVC), the shear elastic modulus depends on the elbow angle, but not at high-level contractions like MVC [11]. This may suggest the involvement of other muscles at higher MVC levels or optimal force production in each condition, as the maximum elbow moment decreases with higher angles, as seen in healthy subjects [11]. Also, in the mild-FSHD group presented here, the elbow moment decreases with increasing angle, aligning with observations in our HG. However, it should also be mentioned that here, due to the experimental paradigm, only mildly affected patients were included, and therefore, the muscle alteration may not have progressed enough to be detected by the SWE, especially in the passive state (see discussion below).

### 4.2. Comparison to a Previously Published Healthy Group

In this pilot study, our primary aim was to investigate the influence of different positions and contraction levels of SWE in mild FSHD. We compared our results with the data collected from healthy adults [11]. Contrary to expectations, the shear elastic modulus did not show prominent differences. We propose two reasons for this discrepancy: disease-specific factors and methodological considerations.

Regarding disease-specific factors, the type and severity of muscular dystrophy could influence the shear elastic modulus [15,16,17,18]. Our study only included patients with an MRC of ≥4− because experimental tasks would not have been feasible for patients below this threshold. Accordingly, half of the FSHD patients exhibited only a minor impact, with no objective weakness in elbow flexion (MRC = 5) but a credible loss of strength. As the MRC is only a subjective parameter, which depends on the investigator, we also compared the elbow moment in every condition. No significant difference was found, which is not surprising given the lack of difference in the shear elastic modulus in the active state. However, for Duchenne dystrophy, higher shear elastic modulus is also described in its early stages in leg muscles [15,16] and for the BB [17], while changes in the muscle are described in some of the patients by magnetic resonance imaging (fatty replacement in the gluteus maximus muscle in three of five Duchenne patients; hyperintensity on STIR images in four of five Duchenne patients) [15]. In contrast, here, five out of eight FSHD patients did not have a pathological Heckmatt score (see Table 2), suggestive of a lack of sufficient structural change in the investigated muscle leading to a normal shear elastic modulus in passive state, as muscle fibrosis could be associated with higher shear elastic modulus and fatty infiltration with lower shear elastic modulus [16,17]. Therefore, disease-specific muscle changes in the investigated type of muscular dystrophy could also influence the resulting shear elastic modulus, as the present FSHD group exhibited a median shear elastic modulus of 3.5 kPa (healthy 4.3 kPa) at 90° and 12.1 kPa (healthy 12.9 kPa) at 180° in the BB. In contrast, previous studies have reported mean shear elastic modulus values of 5.8 kPa (healthy 3.9 kPa) at 90° and 34.9 kPa (healthy 18.9 kPa) at 180° in the BB for Duchenne dystrophy [10] and a median of 25.1 kPa (healthy 18.2 kPa) at 90° for Fukuyama muscular dystrophy [18]. Yet, for other muscle diseases like myositis, decreased shear elastic modulus values are reported [35,36], which decrease with increasing muscle weakness [14,35] and are associated with muscle edema in magnetic resonance imaging [36]. As inflammation in muscle has been repetitively reported in FSHD [37], this might decrease the shear elastic modulus and might cover higher shear elastic modulus due to muscle fibrosis in our mild-FSHD group. Thus, alterations in SWE in a passive state may be present only in severely affected patients (MRC ≤ 3).

Methodologically, it should be noted that—due to the early onset of Duchenne and Fukuyama muscular dystrophy—only children were examined in previous studies, while we presented findings on adults. Furthermore, different shoulder and wrist positions were used in other studies, leading to variations in muscle length and subsequently affecting the shear elastic modulus [23]. More importantly, our measurements were sEMG-controlled. Even low muscle activity can result in higher shear elastic modulus values; thus, sEMG control is recommended [22] but was not always considered in previously published SWE studies on muscle dystrophies. EMG control is crucial for ensuring that measurements during rest accurately represent the passive muscle state and cannot be used if there is no sufficient muscle contraction anymore (MRC 0). Therefore, previous studies on SWE and muscular dystrophy measured the BB during rest but not necessarily during a passive state. However, as we only compared our results to healthy subjects [11], which did not ideally match, there was no matching for age, sex, handedness, and body size. Age especially leads to increased [38,39] and decreased [40,41,42] changes in the shear elastic modulus, which should be considered in future studies.

In summary, we conclude that further studies are needed to compare different muscle diseases (e.g., different muscle dystrophies) and levels of weakness (e.g., MRC 1–5) in well-controlled conditions (e.g., sEMG and exact control of the joint angle) with a larger sample size.

### 4.3. Mean Absolute Percentage Deviation

The FSHD group showed a notable deviation from the elbow moment instructed during the ramp task, as reflected by a higher MAPD than in the HG. However, the MVC itself was not different between both groups. This higher MAPD could indicate a deficit in force control. Therefore, patients seemed to have difficulties “dosing” their muscle activation during the ramp tasks, which could be interpreted as an indicator of central or peripheral changes in the nervous system. Support for this interpretation can be found in other dystrophies, such as Duchenne dystrophy. In Duchenne dystrophy, changes to the muscle spindle [43,44,45] and cortical changes [46,47] have been reported, indicating that dystrophic diseases are not only associated with structural (i.e., dystrophic) changes to the musculature but also with the innervating neural system. In addition, the neural system has been reported to be altered in people with FSHD, for instance, by precentral cortical atrophy [48] and less intracortical inhibition [49]. It could be hypothesized that these neural changes are secondary due to the decreasing muscle mass in people with FSHD [48], potentially leading to poorer control of the remaining muscles. The impaired control could be reflected by a chaotic EMG signal (i.e., variable firing rates), especially at lower contraction intensities, as the adaptation of motor unit recruitment for fine-tuned muscle contractions becomes increasingly difficult [2,50]. Consequently, the higher MAPD in the FSHD group could indicate the pathologically altered neural control reflected in changes in the motor unit and the neural systems due to the disease. Nevertheless, further research is needed to understand the relationships between central and peripheral neural changes and dystrophies.

### 4.4. Strengths and Limitations

This study has several strengths: proper management of (a) muscle strength by the measurement of elbow moment torque, (b) muscle activity by sEMG, and (c) precise joint position. However, there are also some limitations: (a) small sample size with eight patients due to the investigated disease and the inclusion criteria, (b) inclusion of only mildly affected patients due to the inclusion criteria of a minimum MRC score of ≥4−/5 for elbow flexion, ensuring feasible measurement at maximum voluntary contraction (MVC) and during the ramps, (c) no matching of HG for age, sex, handedness, and body size, (d) differences in the exact protocol to minimize the overall length for patients, and (e) alignment with the muscle fiber direction, performed based on visual control.

## 5. Conclusions

The shear elastic modulus in FSHD depends on the joint angle and the percentage of muscle activation. In contrast to other studies, there may be no increase in the shear elastic modulus during the passive state in mild FSHD, although methodological constraints have to be considered. Moreover, our study could reveal an altered force control in mildly affected FSHD patients, which might be related to often-reported subjective muscle weakness. This should be investigated in further studies.

## Figures and Tables

**Table 1:** Demographic and anthropometric information on patients with facioscapulohumeral muscular dystrophy (FSHD) and healthy volunteers.

Parameters	Patients with FSHD	Healthy Volunteers
Sex	3 female, 5 male	7 female, 7 male
Handedness	2 left, 6 right	13 right, 1 both
Age (years)	27/44/52 (24–61)	24/28/31 (22–39)
Body mass index (kg/m^2^)	21.7/26.7/32.6 (21.3–35.2)	21.3/23.1/28.4 (19.4–31.1)
Upper arm circumference (cm)	29/32/35 (27–38)	27/28/30 (26–41)

Age, body mass index, and upper arm circumference are given as 25th/50th/75th percentiles. Minimum and maximum values were provided in parentheses.

**Table 2:** Clinical details of patients with FSHD, including their genetic findings and alterations in the B-mode ultrasound by the modified Heckmatt scale [28].

ID	FSHD Type	Genetic	Weakness According to MRC	Modified Heckmatt Scale	Highest MVC Elbow Flexion Torque in Nm and Respective Elbow Angle	
1	1	D4Z4 repeats of 20 and 18 kb	5/5/5/5/5	1	41.1	120°
2	1	D4Z4 repeats of 33 and 30 kb	5/5/5/4+/4+	2	38.6	120°
3	1	D4Z4 repeats of 36 and 33 kb	4+/4+/4+/5/5	1	43.2	60°
4	1	p13E-11/EcoRI DNA fragments with 15kb/3 Kpn1 copies	4+/4+/4+/4+/4+	3	25.5	90°
5	1	D4Z4 repeat of 37 and 34 kb	4/4−/4+/4/4	1	42.9	120°
6	1	D4Z4 repeats of 30 and 27 kb	5/5/5/5/5	1	66.5	120°
7	1	Abbreviated fragments D4Z4 repeats, exact length not available	4/4−/3/4+/4−	2	22.7	120°
8	1	D4Z4 repeats of 33 and 30 kb	5/5/5/5/5	1	48.2	120°

MRC—Medical Research Council scale (0, 1, 2, 3, 4−, 4, 4+, and 5) [24,25], values for shoulder abduction/internal rotation/external rotation/elbow flexion/extension. Note: The values for EcoRI and Eco+BlnI are mentioned for D4Z4 repeats.

**Table 3:** Dimensions of the biceps brachii (BB) muscle of patients with facioscapulohumeral muscular dystrophy (FSHD).

Elbow Angle	60°	90°	120°	150°	180°	*p*-Value
Length of BB (cm)	12.3/13.5/13.9 (12.0–14.0)	14.0/14.3/15.3 (13.0–15.5)	15.0/16.3/16.7 (14.5–17.0)	15.9/17.3/18.9 (15.2–19.5)	18.3/19.5/21.1 (17.2–21.5)	*<0.001*
CSA of BB (cm^2^)	7.5/9.2/12.1 (7.1–12.9)	7.8/8.7/12.0 (6.4–12.7)	7.4/8.2/11.8 (5.0–12.2)	7.4/8.8/10.9 (4.9–12.4)	7.7/9.2/11.0 (5.3–12.9)	0.531

Data are given as 25th/50th/75th percentiles. Minimum and maximum values were provided in parentheses. BB: biceps brachii muscle. CSA: cross-sectional area. Post hoc test for the length of BB: significant differences between 60° and 150° (*p* = 0.003), between 60° and 180° (*p* < 0.001), as well as between 90° and 180° (*p* = 0.001).

## Data Availability

The data supporting this study’s findings are available upon reasonable request from the authors.

## Appendix A

**Table A1:** The mean absolute percentage deviation (MAPD) of elbow flexion at different elbow angles (60°, 90°, 120°, 150°, and 180°) and at different contraction intensities (at 25%, 50%, and 75% maximum voluntary contraction (MVC)) and the elbow flexion moment at MVC at all elbow angles in the FSHD group and the healthy group (HG).

	60°	90°	120°	150°	180°
FSHD MAPD at 25% MVC (%)	1.5/2.1/2.6	1.6/2.1/2.7	1.7/2.0/3.2	1.5/2.4/4.0	1.7/2.2/2.4
	(1.3–2.9)	(1.1–3.7)	(1.4–6.9)	(1.1–4.7)	(1.4–6.0)
HG MAPD at 25% MVC (%)	1.4/1.7/2.0	1.3/1.4/1.7	1.3/1.5/1.8	1.4/1.5/1.8	1.4/1.7/2.0
	(1.1–2.2)	(1.0–2.0)	(0.9–2.2)	(1.2–1.9)	(1.3–3.1)
FSHD vs. HG					
*p*-value	0.11	*0.01*	*0.029*	*0.035*	0.082
corrected *p*-value	0.157	*0.03*	0.062	0.066	0.137
FSHD MAPD at 50% MVC (%)	2.9/3.6/5.0	2.5/2.7/6.3	2.6/2.9/5.5	2.3/3.4/4.9	2.2/3.0/4.7
	(2.6–11.3)	(1.9–8.6)	(2.3–6.9)	(1.9–6.7)	(1.8–10.0)
HG MAPD at 50% MVC (%)	2.0/2.6/3.0	1.8/2.2/2.4	1.8/2.3/2.3	1.9/2.1/2.9	2.2/2.5/2.9
	(1.6–3.6)	(1.4–3.2)	(1.5–3.3)	(1.5–3.3)	(2.0–3.5)
FSHD vs. HG					
*p*-value	*0.008*	*0.01*	*0.002*	*0.016*	0.238
corrected *p*-value	*0.03*	*0.03*	*0.03*	*0.04*	0.275
FSHD MAPD at 75% MVC (%)	4.4/5.3/6.7	3.1/3.8/5.0	2.7/3.0/5.7	2.7/4.0/5.9	3.6/4.0/7.7
	(2.1–7.2)	(2.0–8.7)	(2.5–6.4)	(2.6–9.9)	(2.9–9.4)
FSHD MAPD at 75% MVC (%)	2.3/3.2/4.3	2.2/3.4/4.1	2.5/2.9/4.1	3.1/4.1/5.2	2.5/3.6/4.2
	(1.9–5.1)	(2.0–4.9)	(2.2–5.1)	(2.5–6.7)	(2.2–5.5)
FSHD vs. HG					
*p*-value	*0.01*	0.127	0.65	0.779	0.115
corrected *p*-value	*0.03*	0.159	0.696	0.779	0.157
FSHD MVC moment (Nm)	26.4/36.6/42.6	27.3/36.0/44.6	26.6/41.3/46.9	21.0/29.6/36.0	19.3/20.1/29.7
	(19.4–62.1)	(18.9–57.6)	(19.9–66.5)	(12.1–42.4)	(11.4–36.9)
HG MVC moment (Nm)	34.6/46.4/63.2	31.0/40.3/54.9	28.0/38.4/55.8	21.0/29.8/43.2	14.9/23.4/40.3
	(28.1–87.1)	(27.0–82.0)	(22.4–76.8)	(15.1–66.3)	(10.4–54.4)

Data are given as 25th/50th/75th percentiles. Minimum and maximum values were provided in parentheses. MAPD: mean absolute percentage deviation; MVC: maximum voluntary contraction; HG: healthy group; FSHD: facioscapulohumeral muscular dystrophy; FSHD vs. HG, *p*-value: comparison between FSHD and HG by the Mann–Whitney U test, corrected *p*-value: corrected for multiple testing using the correction of Benjamini and Hochberg (False Discovery Rate).

**Table A2:** Shear elastic modulus obtained from shear wave elastography (SWE) of the biceps brachii muscle at different elbow angles (60°, 90°, 120°, 150°, and 180°) and at different contraction intensities (passive, maximum voluntary contraction (MVC) and 25%, 50%, and 75% MVC) in FSHD and the healthy group (HG).

	60°	90°	120°	150°	180°
FSHD SWE passive (kPa)	2.7/3.0/3.5	3.4/3.5/5.8	4.6/5.7/10.9	5.9/7.6/11.0	10.0/12.1/13.7
	(2.6–8.4)	(3.3–11.3)	(4.0–17.8)	(5.4–18.7)	(9.6–19.7)
HG SWE passive (kPa)	3.1/3.9/5.1	3.7/4.3/7.6	5.0/6.3/7.6	7.6/9.1/10.8	10.0/12.9/15.5
	(2.6–16.5)	(3.0–12.1)	(3.3–13.5)	(5.6–14.2)	(8.8–16.5)
FSHD vs. HG					
*p*-value	0.05	0.4	0.877	0.416	0.868
corrected *p*-value	0.583	0.8	0.973	0.8	0.973
FSHD SWE at 25% MVC (kPa)	4.7/8.3/17.3	12.5/16.4/22.5	30.8/45.5/63.4	51.5/61.8/67.2	35.1/43.7/46.2
	(4.2–32.3)	(7.7–28.2)	(28.9–71.6)	(48.1–78.9)	(17.1–55.4)
HG SWE at 25% MVC (kPa)	10.1/17.5/21.4	13.2/24.4/30.2	39.5/48.7/59.3	59.3/65.5/75.8	48.8/60.4/69.3
	(5.3–34.0)	(8.3–40.1)	(17.5–83.6)	(42.7–99.5)	(25.9–88.5)
FSHD vs. HG					
*p*-value	0.07	0.161	0.714	0.33	*0.006*
corrected *p*-value	0.583	0.671	0.945	0.8	0.15
FSHD SWE at 50% MVC (kPa)	14.4/38.7/51.2	44.7/53.7/64.1	63.8/71.5/87.5	66.1/71.4/78.9	68.6/70.5/80.3
	(13.2–67.5)	(37.7–73.8)	(56.0–89.0)	(62.4–91.5)	(42.0–85.9)
HG SWE at 50% MVC (kPa)	36.7/40.3/49.5	49.5/62.4/71.2	54.9/74.4/85.7	65.4/76.5/84.0	68.4/78.1/89.0
	(22.1–57.0)	(40.7–85.0)	(40.2–94.0)	(40.0–92.7)	(38.3–94.0)
FSHD vs. HG					
*p*-value	0.57	0.238	0.868	0.616	0.238
corrected *p*-value	0.891	0.744	0.973	0.906	0.744
FSHD SWE at 75% MVC (kPa)	32.4/58.0/73.3	63.1/74.7/78.8	60.1/69.9/88.6	58.4/63.3/68.6	46.3/71.9/79.7
	(28.9–79.9)	(53.1–91.2)	(42.3–89.8)	(58.3–72.6)	(44.8–88.1)
FSHD SWE at 75% MVC (kPa)	53.6/66.2/72.2	58.6/74.8/80.9	61.3/74.7/84.4	55.4/66.6/84.7	60.5/80.1/87.7
	(51.2–81.3)	(50.8–91.9)	(35.6–89.7)	(49.1–101.8)	(48.6–88.6)
FSHD vs. HG					
*p*-value	0.473	0.973	0.967	0.718	0.275
corrected *p*-value	0.845	0.973	0.973	0.945	0.764
FSHD SWE at MVC (kPa)	47.1/63.6/84.0	56.4/71.1/75.3	56.9/65.3/77.5	48.8/53.5/72.9	57.0/67.7/75.3
	(28.1–88.7)	(47.7–77.6)	(30.9–82.6)	(42.1–90.8)	(33.6–76.3)
HG SWE at MVC (kPa)	47.0/60.1/65.2	48.4/53.7/70.4	47.2/53.4/63.0	46.0/57.5/69.0	49.2/52.6/77.6
	(37.7–78.5)	(30.3–78.6)	(43.0–81.4)	(23.0–80.3)	(44.7–82.6)
FSHD vs. HG					
*p*-value	0.374	0.127	0.095	0.92	0.525
corrected *p*-value	0.8	0.635	0.594	0.973	0.875

Data are given as 25th/50th/75th percentiles. Minimum and maximum values were provided in parentheses; SWE: shear wave elastography; MVC: maximum voluntary contraction; HG: healthy group; FSHD: facioscapulohumeral muscular dystrophy. FSHD vs. HG, *p*-value: comparison between FSHD and HG by the Mann–Whitney U test, corrected *p*-value: corrected for multiple testing using the correction of Benjamini and Hochberg (False Discovery Rate).
